# Detection of mammalian orthoreovirus type-3 (Reo-3) infections in mice based on serotype-specific hemagglutination protein sigma-1

**DOI:** 10.1186/s12985-018-1021-8

**Published:** 2018-07-27

**Authors:** Felix Fingas, Daniela Volke, Petra Bielefeldt, Rayk Hassert, Ralf Hoffmann

**Affiliations:** 1 Institute of Bioanalytical Chemistry, Faculty of Chemistry and Mineralogy, Universität Leipzig, Leipzig, Germany; 2 GVG Diagnostics GmbH, Leipzig, Germany; 3 Center for Biotechnology and Biomedicine, Leipzig, Germany

**Keywords:** Mammalian orthoreovirus type-3, FELASA, ELISA

## Abstract

**Background:**

Reovirus type-3 infections cause severe pathologies in young mice and thus influence animal experiments in many ways. Therefore, the Federation of Laboratory Animal Science Associations (FELASA) recommends an annual screening in laboratory mice as part of a thorough health monitoring program. Based on the high protein sequence homology among the different reovirus serotypes, immunofluorescence antibody assay and other indirect methods relying on the whole virus are presumably cross-reactive to antibodies triggered by mammalian orthoreovirus infections independent of the serotype.

**Methods:**

The serotype-specific protein σ-1 was expressed in *Escherichia coli* with an N-terminal Strep-tag and a C-terminal His-tag. The purified Strep-rσ-1-His-construct was used to develop an indirect ELISA by testing defined positive and negative sera obtained by experimental infection of mice as well as field sera.

**Results:**

The Strep-rσ-1-His-ELISA provided high sensitivity and specificity during validation. Notably, a high selectivity was also observed for sera positively tested for other relevant FELASA-listed pathogens. Screening of field samples indicated that a commercial reovirus type-3-based ELISA might be cross-reactive to other murine reovirus serotypes and thus produces false-positive results.

**Conclusions:**

The prevalence of reovirus type-3 might be overestimated in German animal facilities and most likely in other countries as well. The occurrence of other reovirus serotypes, however, raises the question if murine health monitoring programs should be extended to these pathogens.

**Electronic supplementary material:**

The online version of this article (10.1186/s12985-018-1021-8) contains supplementary material, which is available to authorized users.

## Background

Mammalian orthoreoviruses are double-stranded RNA viruses of the *Reoviridae* family that lack lipid envelopes [1]. They consist of a double-capsid structure around a core containing ten genomic RNA-segments [2], RNA-dependent RNA polymerase λ-3 [3], and RNA-associated protein μ-2 [4]. Proteins λ-1 and σ-2 form the inner capsid, while the outer capsid is composed of proteins λ-2, μ-1, σ-3, and σ-1 [5]. Proteins σ-NS, μ-NS, and σ-1s present only in the infected vertebrate host cells [6–9] guide formation of the cytoplasmic inclusion structures [10] and influence hematogenous dissemination [11].

Mammalian orthoreoviruses are divided in four serotypes by considering both the sequence and the antigenic behavior of hemagglutination protein σ-1: prototype strains type-1 (Lang, T1L), type-2 (Jones, T2J), type-3 (Dearing, T3D), and type-4 (Ndelle, T4N) [12–15]. Reovirus type-3 reportedly can infect many species including wild and laboratory mice and thus was included in health monitoring programs [16]. For example, the Federation of Laboratory Animal Science Associations (FELASA) recommends an annual monitoring of reovirus type-3 in mouse and rat colonies [17].

Reovirus T3D is transmitted via fecal-oral and aerosol routes. Infections occur by transcytosis through microfold cells overlaying the bronchus-associated lymphoid tissue in the lung [18] and the Peyer’s patches in the intestine [19]. Thereby, protein σ-1 mediates cell attachment by specific recognition of sialic acid [20, 21] and junctional adhesion molecule-A of the host cell [22] before the virus enters the cell via receptor-mediated endocytosis. Reovirus T3D can further spread to the central nervous system via hematogenous and neural routes [13, 23, 24] leading to lethal encephalitis in suckling mice [13, 15, 25–27] and fatal liver failure in immunodeficient SCID mice [28]. Intranasal infection of adult mice with reovirus T3D results in transient viremia and spreading to multiple organs including the brain. However, clinical symptoms are not observed [29, 30].

Reovirus type-3 infections in laboratory mice facilities are typically detected by serological methods, especially enzyme-linked immunosorbent assay (ELISA) and immunofluorescence antibody assay (IFA) [17], the latter being generally considered as gold standard. Positive tests can be confirmed by virus neutralization (VNT) and hemagglutination-inhibition tests (HI), respectively. However, several reports indicated that both ELISA and VNT based on reovirus T1L, T2J, and T3D, can produce positive results when heterologously challenged with sera of different serotypes [16, 30, 31]. Hence, serotype-specific reovirus T3D detection according to FELASA guidelines is currently challenging and may produce false positive results. Based on an IFA detecting reovirus infections with high sensitivity, we have established an ELISA based on recombinant protein σ-1 to specifically detect antibodies directed against reovirus type-3. This ELISA was highly sensitive, specific and selective when validated with reovirus T3D-positive sera and sera tested positive or negative for other mouse pathogens listed by FELASA. Randomly selected field samples confirmed for a commercial virus-based ELISA that it is cross-reactive towards different reovirus types (false-positive) when screening for reovirus type-3 according to FELASA guidelines.

## Methods

Materials were obtained from the following manufacturers: Amresco (Solon, OH, USA): Yeast extract (bacterial) and tryptone; Applichem (Darmstadt, Germany): Tris(hydroxymethyl)aminomethane (Tris base); BioRad Laboratories GmbH (Munich, Germany): Oriole™ fluorescent gel stain, Precision Plus Protein™ protein standard (unstained and dual color), TransBlot Turbo RTA Transfer Kit LF PVDF Mini; Biosolve BV (Valkenswaard, Netherlands): Acetonitrile (HPLC gradient grade) and formic acid (≥99%); Biozym Scientific GmbH (Hessisch Oldenburg, Germany): Advansta blocking buffer, Advansta washing buffer, and WesternBright™ Sirius substrate solution; Carl Roth GmbH (Karlsruhe, Germany): Ampicillin, glycerol (99.5%), isopropyl β-D-1-thiogalactopyranoside (IPTG), LB medium (Luria/Miller), phosphate-buffered saline pH 7.4 (PBS), PBS with Tween 20 (PBS-T, pH 7.4), sodium dodecyl sulfate (SDS, ≥99.5%), and urea (≥99.5%); Charles River (Wilmington, MA, USA): CD-1 mouse anti-mammalian orthoreovirus type 3 serum (Lot: S1099); GE Healthcare (Fairfield, CT, USA): HisTrap™ HP (1 mL); Jackson ImmunoResearch Laboratories, Inc. (West Grove, PA, USA): Peroxidase-conjugated AffiniPure goat anti-mouse IgG + IgM (H + L, #115062) and anti-rabbit IgG (H + L, #92797); lysozyme (chicken egg white); Merck Millipore (Burlington, MA, USA): anti-reovirus type-3 antibody, σ-1 hemagglutinin, clone 9BG5 (MAB994); NH Dyeagnostics GmbH (Halle, Germany): Immuno Blue HRP Substrate; Roche Diagnostics International AG (Rotkreuz, Switzerland): DNase I (RNase-free); Serva Electrophoresis GmbH (Heidelberg, Germany): Acrylamide/bis(acrylamide) (30% T, 2.67% C), Albumin bovine (Fraction V, protease-free), TEMED, ammonium persulfate (99%), Coomassie® Brilliant Blue G250, glycine (> 98.5%), protease inhibitor mix B, Tween 20 (pure), and trypsin (sequencing grade, MS approved); Sigma-Aldrich GmbH (Taufkirchen, Germany): 2-mercaptoethanol (BioUltra), ammonium bicarbonate (≥99.5%), carbonate-bicarbonate buffer, imidazole (≥99.5%), magnesium chloride hexahydrate (MgCl_2_, ≥99%), and Triton™ X-100; SurModics Inc. (Eden Prairie, Minnesota, USA): StabilZyme Select®, Assay Diluent (Protein-free); Thermo Fisher Scientific (Waltham, Massachusetts, USA): SuperBlock® (PBS), 6×-His Epitope Tag Antibody (HIS.H8); Seramun Diagnostika GmbH (Heidesee, Germany): TMB substrate solution. Water was produced in-house using a Purelab Ultra water purification system (resistance > 18.2 MΩ·cm; total organic content < 5 ppb; ELGA LabWater GmbH, Celle, Germany).

### Preparation of virus stock

Mammalian orthoreovirus type-3 (strain Dearing) and mammalian orthoreovirus type-2 (strain Jones) were prepared by infection of MA-104 cells (ATCC CRL-2378) grown in Dulbecco’s Modified Eagle Medium (DMEM) supplemented with fetal bovine serum (10%), sodium pyruvate (1 mmol/L) and MEM non-essential amino acids (Life Technologies) at a multiplicity of infection (MOI) of 0.5 for three days. Viral stocks were titrated using an indirect immunofluorescence assay indicating a titer of 4.7 × 10^7^ FFU/ml.

### Serum samples

Sera were obtained from experimentally infected animals. Briefly, experimental infections relied on specific-pathogen-free 8 to 10 week old BALB/c (*n* = 15) and C57BL/6 mice (*n* = 15) intranasally infected with mammalian orthoreovirus type-3 strain Dearing with a viral load of 1.2 × 10^6^ FFU (manuscript in preparation). Five mice from both groups were euthanized 2, 4 or 6 weeks post infection and blood was collected by cardiac puncture. Field sera obtained from German animal facilities were analyzed by GVG Diagnostics GmbH.

### Molecular cloning and protein expression

The coding sequence of mammalian orthoreovirus type-3 strain Dearing segment S1 was codon-optimized for *E. coli*, synthesized, and cloned (GenScript, Nanjing, China). A DNA-sequence TGGAGCCACCCGCAATTTGAAAAAGGTGGTAGC (corresponding to WSHPQFEKGGS) coding for Strep-tag II (underlined) was added at the 5′ end to the S1 open reading frame. The construct was cloned into pET21b(+) using restriction sites *NdeI* and *XhoI*. The plasmid was transformed into *E. coli* DH5α for propagation and the nucleotide sequence was confirmed by sequencing (Eurofins, Germany). The plasmid was transformed in *E. coli* Rosetta BL21 (DE3) pLysS. An overnight culture was used to inoculate terrific broth medium (1 L) with ampicillin (100 μg/mL) to obtain a turbidity of 0.1 at 600 nm (OD_600_). Bacterial cultures were grown (continuous shaking, 37 °C) to an OD_600_ of 0.7. The temperature was reduced to 30 °C, and protein expression was induced by addition of IPTG (1 mmol/L). After 4 h, cells were harvested by centrifugation (5000 × *g*, 4 °C, 20 min) and the cell pellet stored at -80 °C until further use.

### Solubilization of inclusion bodies and protein purification

Cells were thawed and suspended in lysis buffer (30 mL, 20 mmol/L Tris-HCl, 3 mmol/L MgCl_2_, pH 8.0). Protease-Inhibitor Mix B (50 μL) and a spatula tip of lysozyme and DNase I were added and the suspension was incubated on ice for 30 min. Cells were lysed on a French® pressure cell press (Thermo Fisher Scientific Inc., Waltham, USA) at 1500 psi and centrifuged (48,000 × *g*, 4 °C, 1 h). The pellet was suspended in PBS (30 mL, 30 min, 4 °C) and centrifuged (15,000 × *g*, 4 °C, 30 min). Resuspension (30 mL) and centrifugation were repeated once with Triton X-100 (2%, v/v) in PBS and twice with urea (1.5 and 3 mol/L) in PBS. The remaining inclusion bodies were dissolved in a solution of urea in PBS (8 mol/L, overnight, 4 °C), centrifuged (48,000 × *g*, 4 °C, 1 h), and adjusted to a final concentration of 25 mmol/L imidazole. The protein solution was sterile-filtered (0.22 μm) and purified by immobilized metal ion affinity chromatography (IMAC) using an ÄKTA purifier FPLC system (GE Healthcare, CT, USA) equipped with a P-900 pump and a P-900 UV detector. Briefly, the HisTrap-column (1 mL, prepacked with Ni Sepharose High Performance) was equilibrated with ten column volumes (CV) of binding buffer (8 mol/L urea, 25 mmol/L imidazole in PBS, pH 7.4) before the protein solution was loaded. Non-binding components were washed out with binding buffer (10× CV) and the protein was eluted with a linear 20 min gradient to 100% elution buffer (8 mol/L urea, 0.5 mol/L imidazole in PBS, pH 7.4). The eluate fraction containing the protein was dialyzed against urea in PBS (4 mol/L, pH 7.4), and stored at -80 °C. Protein concentrations were determined on a NanoDrop 2000c spectrophotometer (Thermo Fisher Scientific, USA) with dialysis buffer as blank.

### SDS-PAGE

Protein samples and fractions were analyzed by sodium dodecyl sulfate-polyacrylamide gel electrophoresis (SDS-PAGE) on a Mini-Protean 3 cell (Bio-Rad Laboratories) using stacking (T = 4%, C = 2.67%) and separation gels (T = 12%, C = 2.67%; 6 cm long) that were 1 mm thick. Samples were diluted (1:4, v/v) with sample buffer (62.5 mmol/L Tris/HCl, pH 6.8, 20% (v/v) glycerol, 2% (w/v) SDS, 5% (v/v) β-mercaptoethanol, 0.5% (w/v) bromophenol blue) to a total volume of 20 μL, heated (5 min, 95 °C), and loaded on the stacking gel. Separation was performed by applying voltages of 100 V for 10 min and 200 V for 45 min using a PowerPac 300 (Bio-Rad Laboratories). Proteins were visualized by colloidal Coomassie Brilliant Blue (CBB) G-250 [32], or Oriole™ fluorescent stain (λ_exc_ = 270 nm, λ_em_ = 604 nm) according to the manufactures protocol. Images were taken on a ChemiDoc MP CCD camera system (Bio-Rad Laboratories).

### Immunoblot

Proteins were electroblotted onto a PVDF membrane using a Trans-Blot Turbo transfer cell (Bio-Rad Laboratories) for 10 min (25 V, 1.3 A, RT). Membranes were blocked with Advan Blocking solution (30 min, RT) and incubated with serum samples (3 μL serum in 10 mL Advan Blocking solution) overnight at 4 °C. Afterwards, membranes were washed two times with Advan Washing solution (5 min), incubated with peroxidase-conjugated goat anti-mouse IgG + IgM (0.5 μL in 10 mL Advan blocking solution plus 0.5% BSA (w/v), 90 min, RT), and washed again with Advan Washing solution (3×, 5 min each). The membrane was incubated with Immuno Blue HRP-Substrate (NH Dyeagnostics, Halle, Germany) for 10 min, washed in Advan washing solution and the fluorescence was recorded (ChemiDoc MP CCD camera system, Bio-Rad Laboratories).

### Enzyme-linked immunosorbent assay (ELISA)

Sera were tested with the Mouse Reovirus type 3 ELISA Kit (XPressBio-ELISA, BioCat, Heidelberg, Germany) according to the manufacturer’s instructions. Briefly, sera were diluted in sample diluent (1:50, v/v) and 0.1 mL were transferred to the wells of a microtiter plate. The plate was covered and incubated (45 min, 37 °C). Wells were washed five times with wash solution (0.3 mL per well). Peroxidase conjugate was added (0.1 mL per well), incubated (45 min, 37 °C), and the wells were washed five times with wash solution (0.3 mL per well). Peroxidase substrate solution containing 2,2′-azino-bis(3-ethylbenzothiazoline-6-sulphonic acid) was added (0.1 mL per well) and incubated (30 min, RT). The absorbance was recorded at 405 nm (Infinite F50 absorbance microplate reader, Tecan, Männedorf, Switzerland). Difference absorbance values were calculated by subtracting the absorbance recorded for a control well.

Alternatively, Medium Bind microplates (Brand, Wertheim, Germany; 96-well, U-shape) were coated with Strep-rσ-1-His (0.1 μg) in carbonate-bicarbonate buffer (50 mmol/L, pH 9.6) overnight at 4 °C. Wells were washed three times (PBS, 300 μL) using a Columbus Pro ELISA washer (Tecan, Männedorf, Switzerland), blocked with Superblock (300 μL, 30 min, RT), and stored at 4 °C. During all incubations, plates were covered with an adhesive foil (SealPlate, Excel Scientific, CA, USA). Wells were incubated with diluted murine serum (1:50 in Assay Diluent; 100 μL, RT) for 45 min. The wells were washed three times with PBS-T (300 μL per well) and conjugate solution was added (100 μL per well, goat anti-mouse-HRP, 1:30,000 in Stabilzyme Select), incubated (30 min, RT), and washed three times, before TMB was added (100 μL per well). After 15 min at RT the reaction was stopped with sulfuric acid (0.5 mol/L, 100 μL per well) and the absorbance recorded at 450 nm.

### Indirect immunofluorescence assay (IFA)

The immunofluorescence assay used acetone-fixed mammalian orthoreovirus type-3-, type-2-, and mock-infected (negative control) MA-104 cells. Mouse serum samples were diluted (1:75) with blocking buffer (5% bovine serum albumin in PBS) and incubated on the cells for 1 h at 37 °C. Cells were washed thoroughly and incubated with Alexa Fluor 488-conjugated goat anti-mouse IgG secondary antibody (1 μg/mL; Thermo Fisher Scientific, MA, USA) and DAPI (final concentration of 0.5 μg/mL) for 1 h at 37 °C. Cells were then washed twice with PBS and images were taken using a fluorescence microscope (IX70, Olympus, Japan).

### Trypsin digest

Gel bands in CBB G-250-stained gels were cut manually or automatically using an EXQuest™ Spot Cutter (Bio-Rad Laboratories), transferred to 0.5 mL polypropylene tubes (Eppendorf, Hamburg, Germany), and destained with aqueous acetonitrile (30%, v/v) containing ammonium bicarbonate (100 μL, 50 mmol/L, w/v) at RT for 5 min. The solution was discarded and the destaining procedure repeated twice before acetonitrile was added. After 5 min, supernatants were discarded and gel pieces were dried on air. A solution of trypsin (Serva Electrophoresis GmbH, 5 μL, 25 ng/μL in 3 mmol/L aqueous ammonium bicarbonate) was added. After incubation (4 h, 37 °C), each solution was transferred to a 0.5 mL polypropylene tube (Eppendorf). Acetonitrile (50 μL) was added and incubated in an ultrasonic bath (Bandelin, Berlin, Germany) for 5 min at RT. The supernatant was transferred to the tube containing the first supernatant and dried in a vacuum concentrator 5301 (Eppendorf) for 1 h at 60 °C. Dried peptides were stored at -20 °C.

### NanoRP-HPLC-ESI-QTOF-MS/MS

Extracted peptides were dissolved in aqueous acetonitrile (20 μL; 3%, v/v) containing formic acid (0.1%, v/v) and analyzed on a Waters nano ACQUITY Ultra Performance Liquid Chromatography (nano UPLC) system coupled online to an electrospray ionization quadrupole time-of-flight mass spectrometer (ESI-QTOF-MS, Synapt G2Si MS, Waters, MS Technologies, Manchester, UK). Samples were loaded on a C_18_-trap column (2G-V/M Trap Symmetry, Waters, 180 μm internal diameter (ID), 2 cm length, and 5 μm particle size) and separated on a C_18_-column (nanoACQUITY UPLC Peptide BEH, 75 μm ID, 10 cm length, and 1.7 μm particle size) at a column temperature of 35 °C using a linear gradient from 3 to 40% aqueous acetonitrile (0.1% formic acid) in 18.5 min and to 85% aqueous acetonitrile (0.1% formic acid) in 3 min. Peptides were identified by data independent acquisition (DIA) in positive ion MS^E^ mode using the following settings: *m/z* 50 to 2000, sampling cone of 40 V, source offset of 80 V, source temperature of 80 °C, cone gas flow of 30 L/h, purge gas flow of 150 mL/h, nanoflow gas pressure of 0.3 bar, and a scan time of 0.5 s. Fragmentation was triggered in the trap before the IMS cell using a collision energy ramp from 18 to 40 V. The doubly protonated signal of Glu-1-Fibrinopeptide B at *m/z* 785.8426 was acquired as lock mass. Acquired data were processed with the Progenesis QI for proteomics software 4.0 (Nonlinear Dynamics), which included Apex3D (version 3.0.14.11) and Peptide3D (version 2.120.0.0) as processing tools, using a lock mass tolerance of 0.254 *m/z* units. Low, elevated and high energy scan counts were individually determined for every sample by the automatic search function of the software. Data were analyzed with the Progenesis QI search engine using the following workflow parameters: database uniprot_sprot (552,259 sequences, downloaded 26.10.2016), precursor and product MHP window (singly protonated peptide mass of the theoretical sequence) was − 1, “number by match for peptide minimum value” was 3, “number peptide for protein minimum value” was 1, “number by match for protein minimum value” was 7, “protein mass maximum atomic mass unit value” was 250,000, false positive rate value was 4, one missed cleavage site, trypsin as “digester reagent”, and methionine oxidation and cysteine carbamidomethylation as variable modifications. The final fragment peptide table generated by the ion accounting output was filtered for proteins from mammalian orthoreovirus 3 type Dearing. Proteins represented by at least three different peptides and identified by “AutoCurate” (> 95% probability) were considered confident.

### ELISA validation and statistical analysis

Repeatability of the indirect ELISA was tested by 36 replicates of positive (PC, anti-His antibody, 1:15,000 in StabilZyme Select®) and negative controls each (NC, Reo-3 [-] serum pool, 1:50 in Assay Diluent). Intermediate precision was tested by five replicates of PC and NC each on five consecutive days. Calculations followed ISO 5725–2 [33, 34]. Assay stability was tested with freshly coated ELISA plates covered with an adhesive foil and stored at 4 °C or 37 °C. All materials needed for testing (controls, buffers, conjugate, TMB, and sulfuric acid) were stored under the same conditions for the same time periods. Three replicates of PC and NC each were tested after storing them at 4 °C or 37 °C for nine time periods up to 200 days.

The limit of detection (LOD) was calculated from the mean absorbance value of sera from mock-infected mice or mice infected with other pathogens (LOD_ref_) plus three times its standard deviation (SD), respectively. D-SN, D-SP and the cut-off values were determined by receiver operating characteristic (ROC) analyses using Graph Pad Prism 7.0 (Graph Pad Software, La Jolla, CA, USA).

### Ethical approval

Animal experiments were approved by the Animal Care and Usage Committee of the Landesdirektion Sachsen, permission no. TVV 04/14.

## Results

**Fig. 1:**
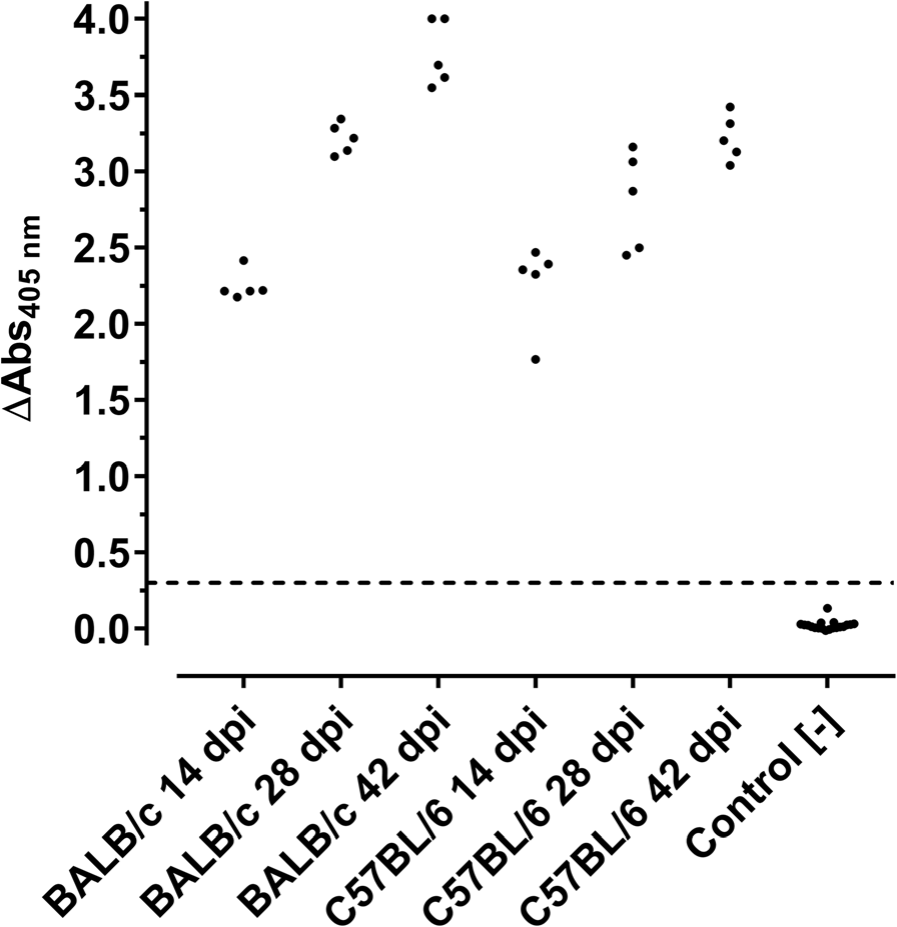
ELISA shows seroconversion of infected mice. Scatter blot of absorbance values obtained by a reovirus T3A-based ELISA of serum collected from BALB/c and C57BL/6 mice two, four, and six weeks after being infected with reovirus T3D and control sera. Test-specific cut-off (0.3) is indicated as a dashed line

FELASA recommends annual routine health monitoring for reovirus type-3 infections in mice, although ELISA tests specific to this virus serotype are not commercially available to the best of our knowledge. To overcome this limitation by developing a sensitive and specific ELISA, 15 C57BL/6 mice and 15 BALB/c mice were infected intranasally by applying a reovirus T3D solution, which simulates the natural infection route (manuscript in preparation). Groups of five animals per strain were sacrificed 14, 28, and 42 dpi, respectively, and blood collected immediately by cardiac puncture. A commercial reovirus type-3 Abney (T3A) ELISA, which does not distinguish reovirus serotypes according to the manufacturer’s supplementary sheet, clearly confirmed all serum samples of both mouse strains as seropositive (Fig. 1).

**Fig. 2:**
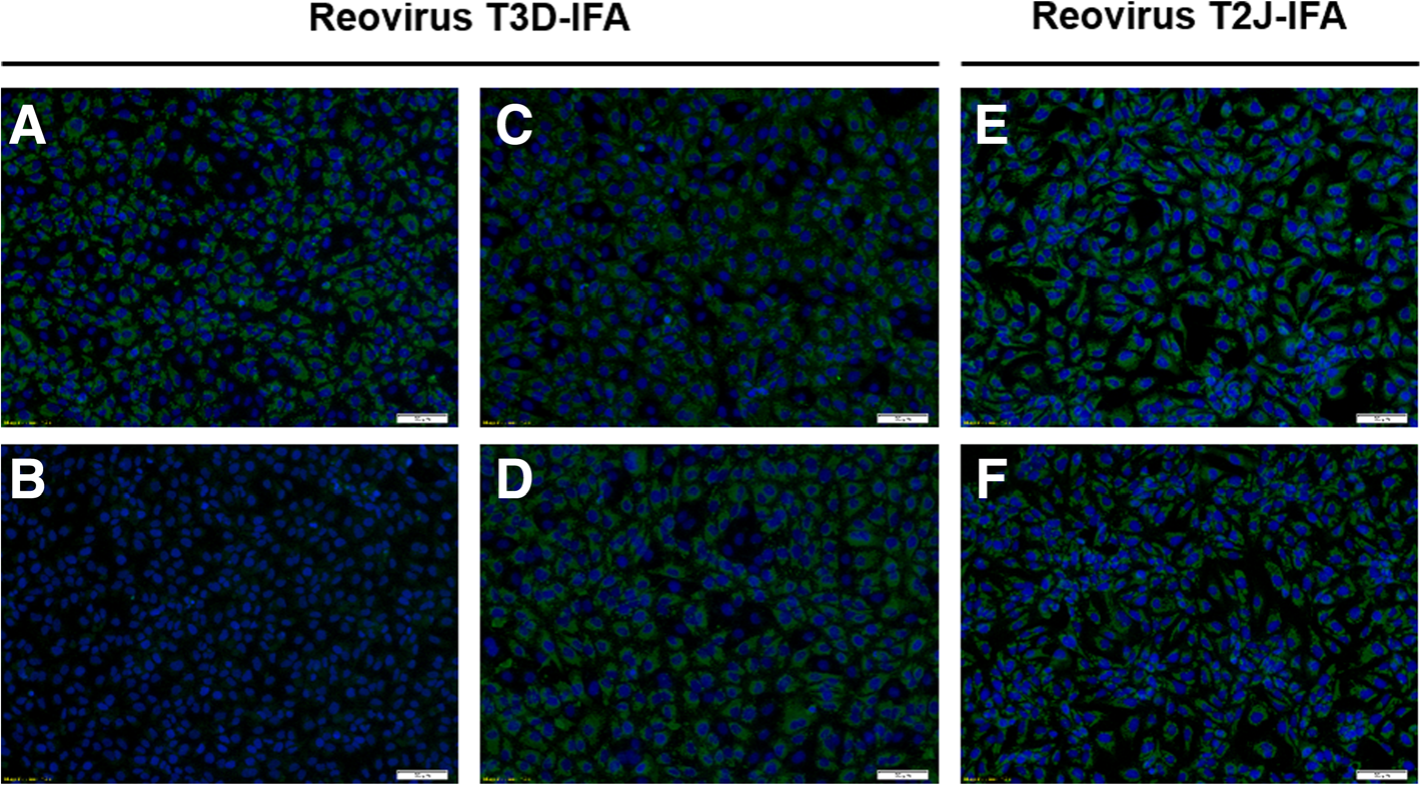
IFA confirms seroconversion of reovirus T3D-infected mice. IFA based on reovirus T3D- (**a**-**d**) and reovirus T2J-infected (**e** + **f**) MA104-cells probed with anti-reovirus type-3 σ-1 monoclonal antibody (mAb, **a**; positive control), serum of an uninfected specific pathogen-free (SPF) mouse (**b**; negative control) and sera of reovirus T3D-infected BALB/c (**c** + **e**) and C57BL/6 (**d** + **f**) mice. Fluorescence staining relied on an Alexa Fluor 488-conjugated goat anti-mouse IgG antibody (green). Cell nuclei were stained with DAPI (blue). Scale bar: 50 μm

The absorbance values of the ELISA ranged from 1.7 to 4.0 and mean values of the groups from 2.2 ± 0.1 (BALB/c; 14 dpi) to 3.8 ± 0.2 (BALB/c; 42 dpi), i.e., at least fivefold above the cut-off. The absorbance values determined in the ELISA increased for both mouse strains over the infection period of six weeks. Expectedly, the blood samples collected two weeks post infection were strongly positive showing a rapid and robust immune response. Additionally, sera were confirmed by IFA based on reovirus T3D-infected MA104-cells. (Fig. 2 a-d).

All 30 sera showed a typical, reovirus-specific cytoplasmic pattern in infected cells, whereas no specific signals were identified in uninfected cells, showing the high sensitivity and specificity of the established IFA. However, an IFA using reovirus T2J-infected MA104-cells (Fig. 2, e-f) was also positive with an indistinguishable staining pattern indicating that the sera derived from reovirus T3D-infections are cross-reactive to reovirus T2J, which is in full agreement with the literature [30, 31] and the high protein sequence homology among reovirus types 1–3, i.e., at least 80% and mostly > 90% identity [35]. Only proteins σ-1 and σ-1s are less conserved with an σ-1 identity of 27% (T3D vs. T1L) and 24% (T3D vs. T2J), whereas σ-1 proteins of type-3 strains Dearing and Abney are 99% identical (Additional file 1). Correspondingly, the immuno-stained cytoplasmic patterns of T2J- and T3D-IFA were identical within the experimental reproducibility and thus were unable to define the serotype.

**Fig. 3:**
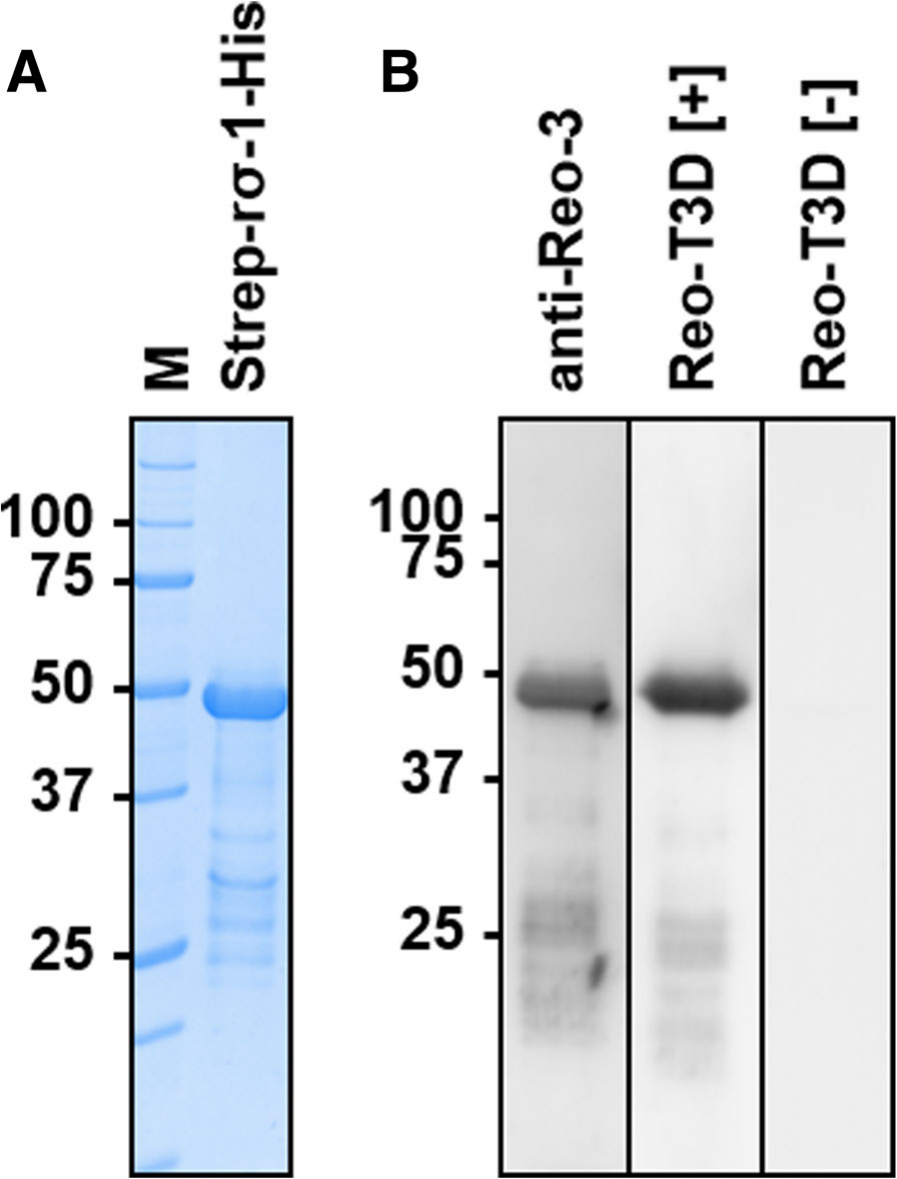
SDS-PAGE (**a**) and immunoblots (**b**) of recombinant Strep-rσ-1-His after purification from inclusion bodies. **a** Proteins were stained with colloidal Coomassie. M denotes marker proteins with the molecular masses in kDa indicated left. **b** Electroblot probed with a commercial anti-reovirus type-3 serum (anti-Reo-3), serum obtained from a mouse experimentally infected with reovirus T3D (Reo-T3D [+]), and a serum from an uninfected mouse (Reo-T3D [-])

Hence, cell attachment protein σ-1 was expressed in *E. coli* with an N-terminal Strep-tag and a C-terminal His-tag. Strep-rσ-1-His (51.6 kDa) was present mostly in inclusion bodies and thus purified by IMAC after solubilization in 8 mol/L urea buffer. The protein fraction (8.5 mg protein per liter cell culture) showed only one major band in SDS-PAGE at an apparent molecular weight of ~ 49 kDa besides a few faint bands indicating minor impurities (Fig. 3a and Additional file 2). The identity of the main band was confirmed after in-gel digestion by tandem mass spectrometry as σ-1 (protein score > 18,000, sequence coverage of 54%, Additional file 3). When electroblotted and probed with a commercial anti-reovirus type-3 serum (anti-Reo-3) and sera from infected mice (Reo-T3D [+]) the Strep-rσ-1-His band was strongly detected, whereas sera from uninfected mice (Reo-T3D [-]) did not stain this band (Fig. 3b). Reconstitution of a folded protein state by stepwise reduction of urea concentration via dialysis was not successful, as precipitation occurred at urea concentrations below 4 mol/L.

**Fig. 4:**
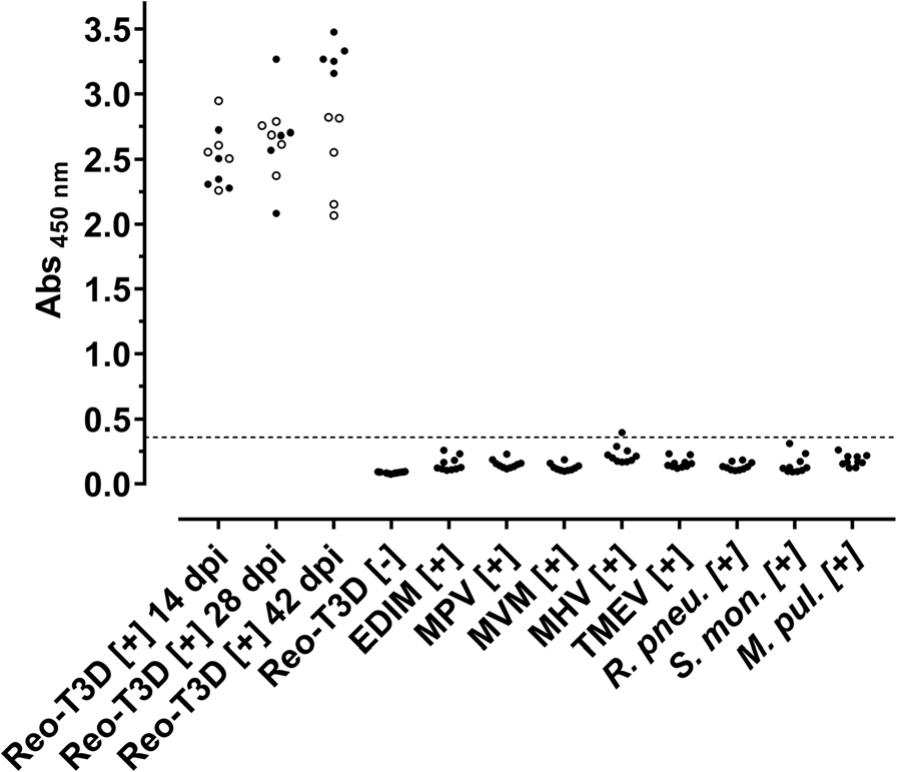
Strep-rσ-1-His-based indirect ELISA is highly sensitive, specific and selective. Scatter blot of absorbance values recorded for reovirus T3D-infected BALB/c (filled circles, *n* = 15) and C57BL/6 (open circles, *n* = 15) mice, sera obtained from uninfected SPF mice (*n* = 30), and sera positive for other viral and bacterial FELASA-listed pathogens (*n* = 10 each). Cut-off value defined by ROC analysis (0.36) is indicated as dashed line

An indirect ELISA was established with purified Strep-rσ-1-His and several critical parameters of the test conditions were optimized, such as plate type, coating conditions, buffer composition, antigen load, and antibody concentration. When the final protocol was applied to all 30 sera of experimentally infected mice, they were strongly positive with absorbance values ranging from 2.1 to 3.5 (Fig. 4). The mean values slightly increased with the infection period from 2.5 ± 0.2 at 14 dpi to 2.9 ± 0.5 at 42 dpi. At 42 dpi, BALB/c mice showed a mean absorbance value of 3.3 ± 0.1 and C57BL/6 a lower value of 2.5 ± 0.3. The readout of the commercial anti-reovirus type-3 serum was 2.4 (data not shown). In contrast, 30 sera obtained from SPF control mice were all negative with a mean value of 0.09 ± 0.01. Notably, sera positive for eight other viral and bacterial FELASA-listed pathogens, which were invariably obtained by experimental infections and confirmed seropositive by ELISA and partially IFA for the corresponding infectant (data not shown), were not cross-reactive with all 80 absorbance values below 0.4 (mean absorbance value of 0.16 ± 0.06), i.e., mouse rotavirus (EDIM), mouse parvovirus (MPV), minute virus of mice (MVM), mouse hepatitis virus (MHV), Theiler’s murine encephalomyelitis virus (TMEV), *Rodentibacter pneumotropicus* (*R. pneum.*, prev. *Pasteurella pneumotropica* Biovar Jawetz [36]), *Streptobacillus moniliformis* (*S. mon.*), and *Mycoplasma pulmonis* (*M. pul.*).

The LOD_ref_ (Mean + 3× standard deviation) was 0.31 considering in total 110 reovirus T3D- defined negative sera, whereas a ROC analysis of all positive and negative sera suggested a minimal cut-off of 0.36 providing 100% diagnostic sensitivity (D-SN) and 99.1% diagnostic specificity (D-SP). For all further analyses, a cut-off of 0.40 was applied providing a D-SN and a D-SP of 100% for the test set. Further parameters resulting from validation of the immunoassay are provided in Table 1 and assay stability is shown in additional file 4.

**Table 1:** Diagnostic parameter of Strep-rσ-1-His indirect ELISA

Diagnostic parameter	Value
Repeatability	PC: 5.4% CoV (*n* = 36) NC: 6.0% CoV (*n* = 36)
Intermediate Precision	PC: 11.2% CoV (*n* = 5) NC: 15.8% CoV (*n* = 5)
Long-term Stability	> 70 d (4 °C); < 10 d (37 °C)
LOD_ref_ (D-SN; D-SP)	0.31 (100%, *n* = 30; 98.2%, *n* = 110)
ROC_ref_ (D-SN; D-SP)	0.36 (100%, *n* = 30; 99.1%, *n* = 110)
Selectivity based on ROC_ref_	98.8% (*n* = 80)

Raw data of repeatability, intermediate precision, and long-term stability are listed in the supplement (Additional file 5). *Ref* Calculation based on sera of experimentally infected and control SPF mice, *PC* positive control, *NC* negative control, *CoV* coefficient of variation

**Fig. 5:**
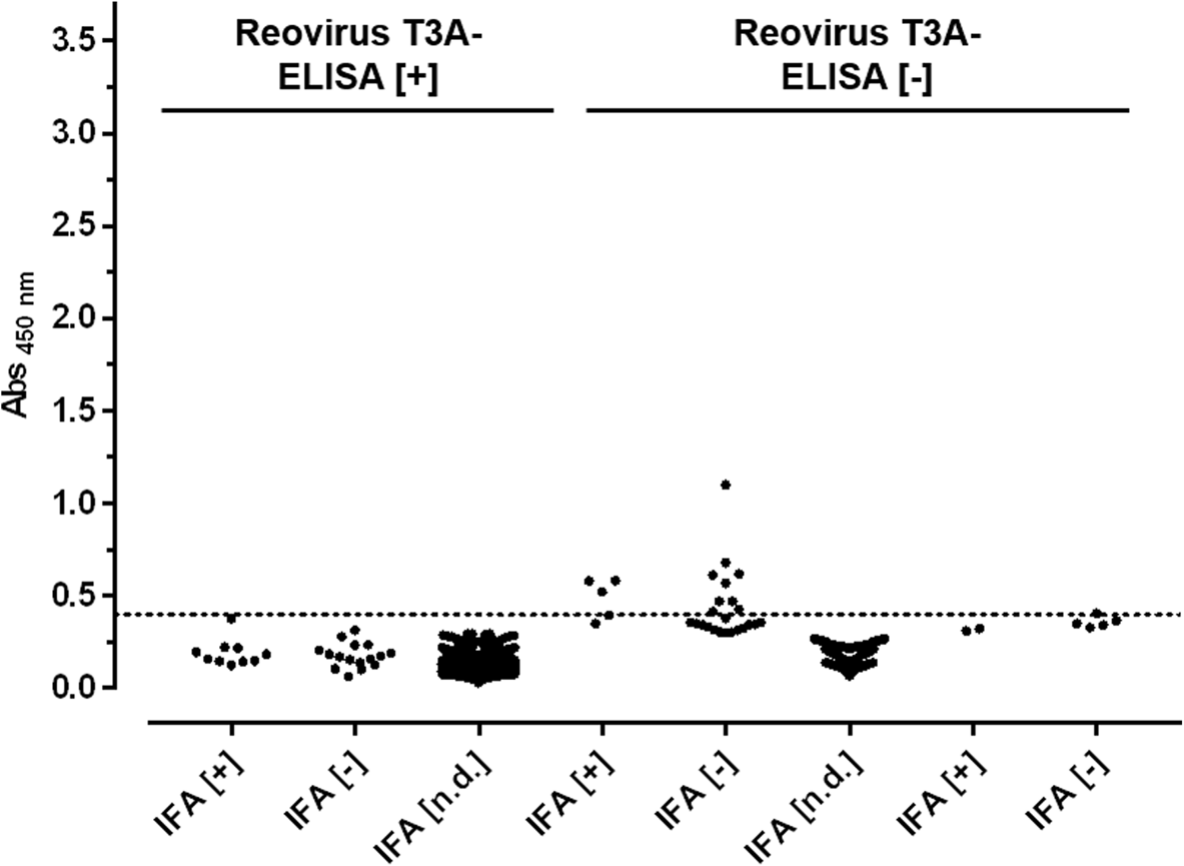
Screening of field sera with Strep-rσ-1-His-ELISA. Scatter plot of absorbance values recorded at 450 nm (Abs _450 nm_) using randomly collected sera from an in-house database that were partly previously tested with a commercial reovirus T3A-based ELISA. Sera are sorted according to the results of T3D-IFA. Cut-off value (0.4) is indicated as dashed line

Finally, 852 field sera obtained from different German animal facilities and previously tested with the commercial reovirus type-3 Abney (T3A) ELISA were probed in the Strep-rσ-1-His ELISA (Fig. 5). Surprisingly, the mean value of 26 sera previously tested positive was 0.18 ± 0.07 with one serum slightly below the cut-off despite the high sensitivity of the Strep-rσ-1-His-ELISA obtained for both mouse strains in the experimental infection study. Further analysis by reovirus T3D-IFA confirmed this and nine more sera (38.5%; 10 of 26) as seropositive whereas the other 16 sera (61.5%) were clearly negative (data not shown). A total of 16 sera (8 positive, 8 negative) were present in sufficient amounts to perform a T2J-IFA. All eight T3D-IFA [+] sera were tested positive whereas of the eight T3D-IFA [-] sera, two were tested positive and six confirmed negative (data not shown). IFA- and Strep-rσ-1-His-ELISA negative sera that were tested positive by the commercial virus-based ELISA were positive for up to six other pathogens when applied to other ELISA tests from the same manufacturer. Thus, only ten of the previously 26 identified reovirus infections could be confirmed as reovirus-positive. According to our results, none of them is positive for reovirus type-3. The mean absorbance value of the 826 sera previously tested negative was 0.14 ± 0.08, i.e., at the background level. All sera with an absorbance value > 0.30 (*n* = 28) were checked by T3D-IFA identifying five sera as positive. Assuming that sera tested negative by both ELISA are true negatives, the specificity of the Strep-rσ-1-His-ELISA with field sera is 98.8% (811/821).

## Discussion

Mammalian orthoreovirus type-3 reportedly has a very low serological prevalence of 0.01–0.05% in laboratory mice [37, 38], whereas the serological prevalence of reovirus type-3 in adult humans exceeds 75% with acute infections peaking in young adults [39]. Considering furthermore the high contamination rate of biological materials [40, 41] and the resistance of the non-enveloped virus to chemical and physical treatments [42], there is a continuous risk of reovirus infections in laboratory mice. Undetected infections can spread causing severe pathologies influencing experiments in different ways [16]. Hence, screening for reovirus type-3 infections in breeding colonies is an important part of health monitoring.

The low prevalence in combination with the lack of commercial anti-reovirus type-3 sera, illustrates the challenge of establishing a diagnostic test. Thus, BALB/c and C57BL/6 mice were experimentally infected with reovirus T3D and seroconversion was confirmed by ELISA and IFA. The chosen mice strains represent common inbred strains widely used in basic and applied research. As already reported for virus-based ELISA and virus neutralization test [30, 31], the reovirus T3D sera were also cross reactive to T2L-infected cells using an IFA established in-house. Hence, the IFA appears to be a highly sensitive method for indirect detection of reovirus infections, but does not allow subtyping the serotypes due to the high sequence homologies that often exceeds 90% for a given protein [35]. Only the gene segment S1 determining the neurovirulence [14] and encoding σ-1s and spike-forming hemagglutination protein σ-1 differ significantly among all three major serotypes. Lee et al. demonstrated that mAbs raised against σ-1 of serotype T3D are not cross-reactive to σ-1 of serotypes T1L and T2D, whereas mAbs directed against other virus proteins, such as λ-2, μ-1, μ-NS, σ-2, σ-3, and σ-NS, where cross-reactive [43]. Thus, σ-1 was recombinantly expressed in *E. coli* with an N-terminal Strep-tag and a C-terminal His-tag. The obtained Strep-rσ-1-His was pure, but the natural trimeric structure of σ-1 could not be reconstituted, probably due to the highly hydrophobic N-terminal part that normally anchors the protein in the virion [44]. Truncation of this N-terminal sequence or fusion to GST may provide a soluble trimeric structure resembling the native structure more closely [21, 45, 46]. However, Chappell et al. reported carbohydrate-binding domains in the N-terminal tail region [20], which may represent important epitopes that might be masked by a GST-tag.

Thus, full-length σ-1 was expressed and the Strep-rσ-1-His-based ELISA showed very high sensitivity and specificity (99%) when tested with defined positive and negative sera obtained from the experimental infection study. Similar results have been reported for a protein-based ELISA detecting the avian reovirus [47–49]. The ELISA protocol established here is fast (< 2 h) and reliable enabling high throughput screenings. The low sample volume of only 2 μL of serum allows direct blood sampling in mice avoiding sentinel animals and thus reducing the number of mice kept in breeding colonies. Remarkably, the Strep-rσ-1-His-based ELISA was not cross-reactive to defined sera positive for other FELASA-listed pathogens confirming its high selectivity. Surprisingly, only ~ 40% (10/26) of the sera obtained from different German animal facilities previously tested positive by the commercial virus-based ELISA were confirmed as reovirus positive by the in-house IFA, whereas all were negative in the Strep-rσ-1-His-ELISA (cut-off 0.4) indicating that the mice were not infected by reovirus type-3. This raises the question, if σ-1 is indeed the best choice for establishing an ELISA. Because of its important function in cell attachment and infection, σ-1 appears to be an effective target for the immune system. Indeed, Major et al. reported σ-1 of reovirus T1L as a dominant antigen in mice independent of the route of infection, genetic background, and antibody subclass [50]. Thus, an σ-1 independent immune response producing false-negative results appears to be unlikely.

Further serotype characterization of positively tested sera by a hemagglutination assay was impossible due to the low volumes of sera available. Moreover, the infection status of field sera could not be verified by electron microscopy, sandwich ELISA or PCR [51–53] due to the lack of intestine and fecal samples. The latter assays are also limited to the transient infection period as the virus is cleared from the intestine within seven days post infection in immunocompetent adult mice [54]. Thus, the final rating of the pretested field sera is challenging, as important parameters are unknown, i.e., genetic background, breed, sex, treatment, sentinel-status, diet and, environmental conditions. Likewise, the defined sera used for assay validation do not represent the entire target population and, hence, only present a random sample.

Taken together, serotype cross-reactivity of virus-based ELISA and IFA are likely due to the high sequence homologies. This may trigger false-positive diagnoses and overestimated prevalence rates of reovirus type-3 infections, at least in German animal facilities, which needs to be further examined. Infections with reovirus type-1 and type-2 generally proceed milder [30], but the induced immune response will also influence animal experiments. Therefore, reovirus type-1 and type-2 should eventually be incorporated in annual murine health monitoring programs.

## Conclusion

An ELISA for the detection of murine reovirus type-3 infections based on Strep-rσ-1-His provided favorable sensitivity, specificity, and selectivity when validated with defined positive and negative sera obtained from experimentally infected mice. Current assays relying on the whole reovirus, such as IFA and a commercial ELISA, are not serotype specific and thus can incorrectly identify reovirus type-3 infections. The assay established here uses a single recombinant protein of reovirus type-3 with low sequence homologies to the other major serotypes. The fast assay protocol using only 2 μL of serum enables high-throughput screening and can be easily adopted to other animal species. The presented data indicate that the prevalence of reovirus type-3 infections in German animal facilities is lower than assumed, as infections with other reovirus serotypes have generated false positive results.

## Additional files

Additional file 1: Alignment of σ-1 amino acid sequences shows conservation within and diversity across reovirus serotypes. The following GenBank accession numbers were used for the BLASTp alignment: HM159619.1 (T3D), GU589583.1 (T3A), M35964.1 (T2J) and M35963.1 (T1L). Absolute and percentage values of identity, positives and gaps as well as percentage of query cover are provided. Information modified after [36]. (XLSX 11 kb)

Additional file 2: SDS-PAGE and immunoblots of protein preparations and fractions obtained during expression and purification of Strep-rσ1-His**.** (A) Expression of Strep-rσ-1-His in *E. coli*. The following samples were loaded: cell extract of *E. coli* without inducing protein expression (0 h), bacterial pellets after 1 h and 2 h, and cytoplasm (Cyt) and inclusion bodies (IBs) prepared from cells 4 h after the expression was induced by IPTG. (B) Western Blot of (A) probed with an anti-His mAb. Affinity-chromatographic purification of the cytoplasmic fraction did not yield in detectable amounts of Strep-rσ-1-His (data not shown). (C) Wash fractions 1 to 4 (W1-W4) obtained during solubilization of inclusion bodies, and the solubilized protein in the obtained solution (Sol). Proteins were stained with colloidal CBB G-250. M denotes marker proteins with the molecular masses in kDa indicated left. (TIF 246 kb)

Additional file 3: Identity confirmation of Strep-rσ-1-His by nanoRP-UPLC-ESI-QTOF-MS/MS. Purified proteins was separated by SDS-PAGE and digested with trypsin. Given are the peptide sequences identified, the corresponding protein score, protein false-positive rate. (XLSX 12 kb)

Additional file 4: Temperature-dependent stability of Strep-rσ-1-His-ELISA. Freshly coated ELISA plates were stored at 4 °C (black) and 37 °C (red), respectively, including all material needed for testing (controls, buffers, conjugate, substrate, sulfuric acid). Positive control (solid line, anti-His antibody) and negative control (dashed line, pool of reovirus type-3 [−] sera) were assayed in replicates of three at a total of nine time points for each storage temperature. Mean values and standard deviations are indicated. (TIF 178 kb)

Additional file 5: Excel Sheet Raw data of ELISA validation. Sheet 1: Raw data of repeatability, intermediate precision and calculation of the limit of detection. Sheet 2: Raw data of stability experiments. Sheet 3: Raw data of ROC analysis. (XLSX 21 kb)

## Abbreviations

CoV: Coefficient of variation
D-SN: Diagnostic sensitivity
D-SP: Diagnostic specificity
*E. coli*: *Escherichia coli*
EDIM: Epizootic diarrhea of infant mice (mouse rotavirus)
ELISA: Enzyme-linked immunosorbent assay
FELASA: Federation of Laboratory Animal Science Associations
FFU: Fluorescence-forming units
HI: Hemagglutination-inhibition test
IFA: Immunofluorescence antibody assay
IMAC: Immobilized metal ion affinity chromatography
LOD: Limit of detection
mAb: Monoclonal antibody
*M. pul.*: *Mycoplasma pulmonis*
MHV: Mouse hepatitis virus
MPV: Mouse parvovirus
MVM: Minute virus of mice
*R. pneu.*: *Rodentibacter pneumotropicus*
ROC: Receiver operating characteristics
*S. mon.*: *Streptobacillus moniliformis*
SCID: Severe combined immunodeficiency
SDS-PAGE: Sodium dodecyl sulfate polyacrylamide gel electrophoresis
SPF: Specific pathogen-free
T1L: Type-1 Lang
T2J: Type-2 Jones
T3A: Type-3 Abney
T3D: Type-3 Dearing
T4N: Type-4 Ndelle
TMEV: Theiler’s murine encephalomyelitis virus
VNT: Virus neutralization test
