# Supplementary figures and images for: Detection of mammalian orthoreovirus type-3 (Reo-3) infections in mice based on serotype-specific hemagglutination protein sigma-1
Source: Virol J. 2018 Jul 27;15:114. doi: 10.1186/s12985-018-1021-8 (PMC6062942; doi:10.1186/s12985-018-1021-8)

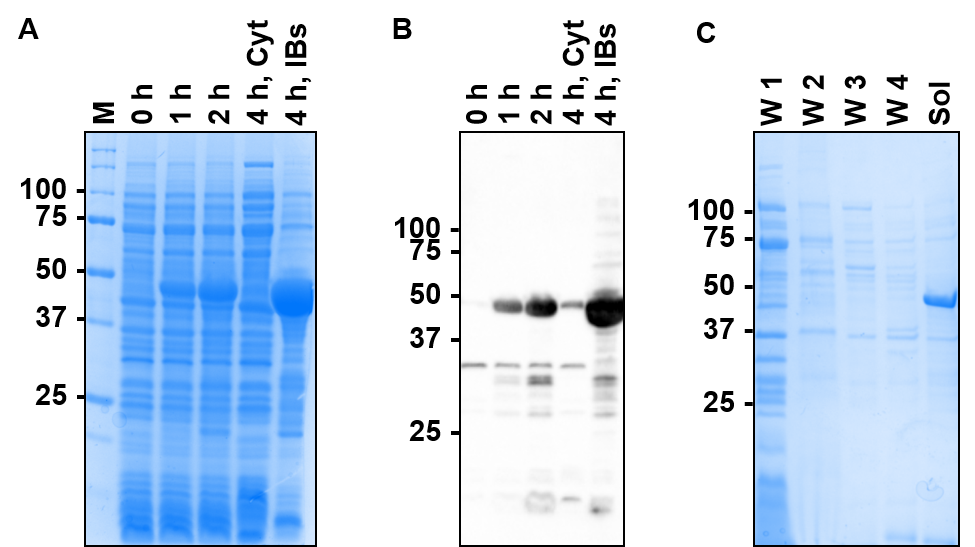

Supplement: Supplementary file 2 — SDS-PAGE and immunoblots of protein preparations and fractions obtained during expression and purification of Strep-rσ1-His. (A) Expression of Strep-rσ-1-His in E. coli. The following samples were loaded: cell extract of E. coli without inducing protein expression (0 h), bacterial pellets after 1 h and 2 h, and cytoplasm (Cyt) and inclusion bodies (IBs) prepared from cells 4 h after the expression was induced by IPTG. (B) Western Blot of (A) probed with an anti-His mAb. Affinity-chromatographic purification of the cytoplasmic fraction did not yield in detectable amounts of Strep-rσ-1-His (data not shown). (C) Wash fractions 1 to 4 (W1-W4) obtained during solubilization of inclusion bodies, and the solubilized protein in the obtained solution (Sol). Proteins were stained with colloidal CBB G-250. M denotes marker proteins with the molecular masses in kDa indicated left. (TIF 246 kb) [file 12985_2018_1021_MOESM2_ESM.tif]

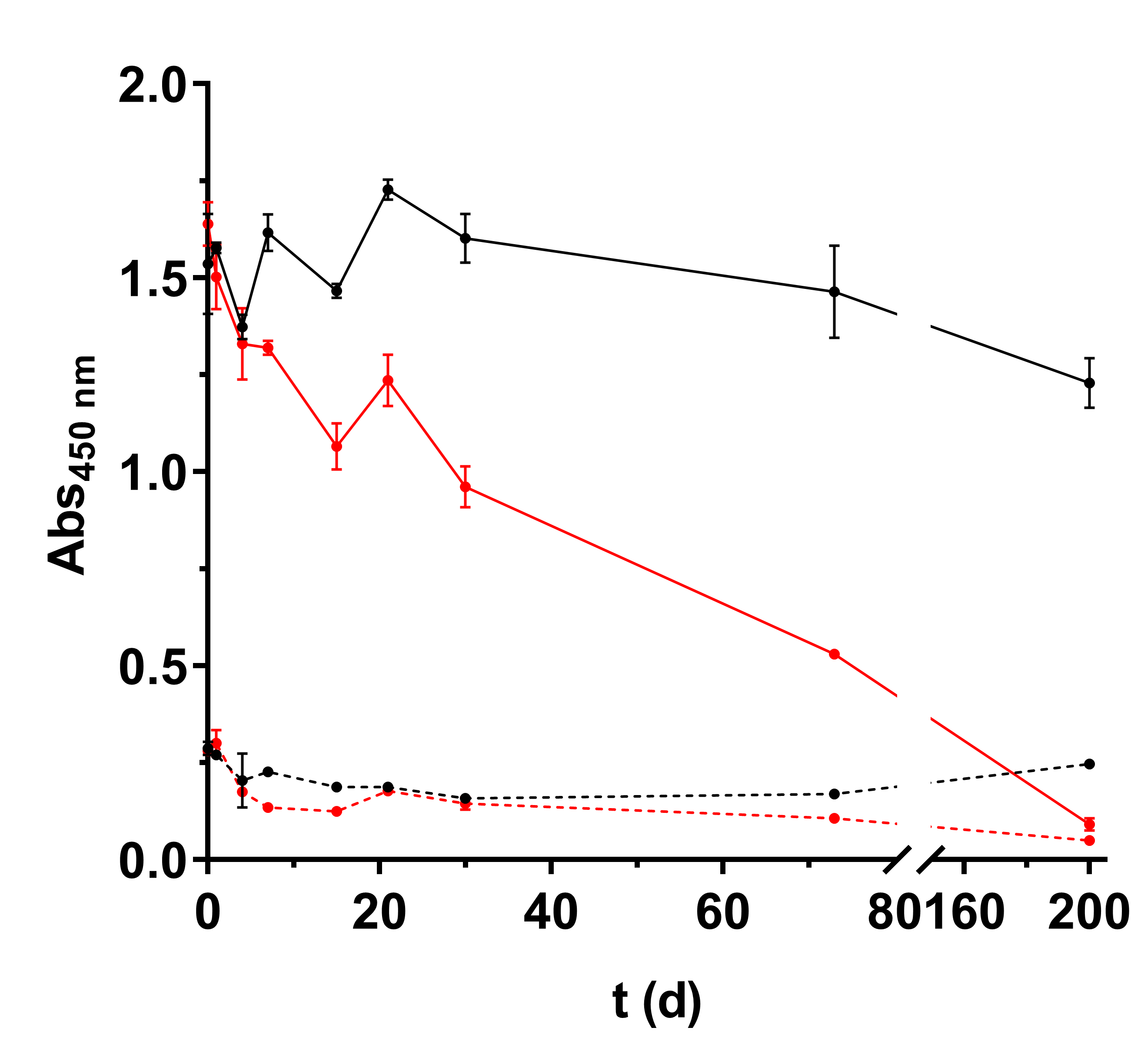

Supplement: Supplementary file 4 — Temperature-dependent stability of Strep-rσ-1-His-ELISA. Freshly coated ELISA plates were stored at 4 °C (black) and 37 °C (red), respectively, including all material needed for testing (controls, buffers, conjugate, substrate, sulfuric acid). Positive control (solid line, anti-His antibody) and negative control (dashed line, pool of reovirus type-3 [−] sera) were assayed in replicates of three at a total of nine time points for each storage temperature. Mean values and standard deviations are indicated. (TIF 178 kb) [file 12985_2018_1021_MOESM4_ESM.tif]
